# Development of a multi-component intervention to promote participation of Black and Latinx individuals in biomedical research

**DOI:** 10.1017/cts.2021.797

**Published:** 2021-06-14

**Authors:** Maria I. Danila, Jeroan J. Allison, Karin Valentine Goins, Germán Chiriboga, Melissa Fischer, Melissa Puliafico, Amy S. Mudano, Elizabeth J. Rahn, Jeanne Merchant, Colleen E. Lawrence, Leah Dunkel, Tiffany Israel, Bruce Barton, Fred Jenoure, Tiffany Alexander, Danny Cruz, Marva Douglas, Jacqueline Sims, Al Richmond, Erik D. Roberson, Carol Chambless, Paul A. Harris, Kenneth G. Saag, Stephenie C. Lemon

**Affiliations:** 1 Department of Medicine, Division of Clinical Immunology/Rheumatology, University of Alabama at Birmingham School of Medicine, Birmingham, AL, USA; 2 University of Alabama at Birmingham Center for Clinical and Translational Science, University of Alabama at Birmingham School of Medicine, Birmingham, AL, USA; 3 UMass Center for Clinical and Translational Science, University of Massachusetts Medical School, Worcester, MA, USA; 4 Department of Population and Quantitative Health Sciences, University of Massachusetts Medical School, Worcester, MA, USA; 5 Department of Medicine, University of Massachusetts Medical School, Worcester, MA, USA; 6 Vanderbilt Institute for Clinical and Translational Research (VICTR), Vanderbilt University Medical Center, Nashville, TN, USA; 7 Department of Biomedical Informatics, Vanderbilt University Medical Center, Nashville, TN, USA; 8 Community Campus Partnerships for Health, Raleigh, NC, USA; 9 Department of Neurology, University of Alabama at Birmingham School of Medicine, Birmingham, AL, USA

**Keywords:** Research participation, health disparities, eConsent, intervention

## Abstract

**Introduction:**

Barriers to research participation by racial and ethnic minority group members are multi-factorial, stem from historical social injustices and occur at participant, research team, and research process levels. The informed consent procedure is a key component of the research process and represents an opportunity to address these barriers. This manuscript describes the development of the Strengthening Translational Research in Diverse Enrollment (STRIDE) intervention, which aims to improve research participation by individuals from underrepresented groups.

**Methods:**

We used a community-engaged approach to develop an integrated, culturally, and literacy-sensitive, multi-component intervention that addresses barriers to research participation during the informed consent process. This approach involved having Community Investigators participate in intervention development activities and using community engagement studios and other methods to get feedback from community members on intervention components.

**Results:**

The STRIDE intervention has three components: a simulation-based training program directed toward clinical study research assistants that emphasizes cultural competency and communication skills for assisting in the informed consent process, an electronic consent (eConsent) framework designed to improve health-related research material comprehension and relevance, and a “storytelling” intervention in which prior research participants from diverse backgrounds share their experiences delivered via video vignettes during the consent process.

**Conclusions:**

The community engaged development approach resulted in a multi-component intervention that addresses known barriers to research participation and can be integrated into the consent process of research studies. Results of an ongoing study will determine its effectiveness at increasing diversity among research participants.

## Introduction

Maximizing population benefits of scientific discoveries requires participation in health-related research studies by a diverse group of individuals, including members of specific sub-populations who experience health inequities, such as African American and Latinx minority groups. Although minority populations living in the USA disproportionately suffer from the leading causes of death and disability [1], they are often underrepresented [2] in health-related research compared to non-Latinx whites. This underrepresentation in clinical studies has resulted in treatment guidelines and clinical practices that may not be equally effective across diverse racial/ethnic groups, potentially further perpetuating health inequalities [3].

Critical challenges exist in the recruitment and retention of diverse participants in health-related research studies. In African American and Latinx communities, there is a legacy of mistrust stemming from historical injustices in biomedical research, such as the infamous Tuskegee and Guatemalan studies conducted in persons with sexually transmitted diseases [4], including perceptions of conspiracy to harm and concerns related to misuse of individual data [5]. Research team barriers include persistent stereotypes that minority participants are more difficult to reach and less compliant to study procedures once enrolled, portraying information in an unnecessarily complex and insufficient manner, and a lack awareness of the barriers to research participation experienced by racial and ethnic minorities, such as mistrust, and of the impact of their own words and behaviors on these individuals [6–9]. Potential participants may also experience limited research literacy, defined as “the capacity to obtain, process, understand, and act on basic information needed to make informed decisions about research participation” [10] including perceptions that research primarily benefits others [11–17].

Informed consent is the core initial component of the health-related research process that assists potential participants in decision-making around enrolling in a research study. Through informed consent, investigators aim to provide clear and accurate information on study purpose, salient aspects of study involvement, and potential study-specific risks and benefits. However, informed consent procedures have become increasingly regulated, are in many cases onerous, and often serve the interests of research institutions and funders rather than meeting the needs of prospective trial participants [18]. Research participants frequently remain misinformed or underinformed about the goals of the research project or its risks and benefits [10,11,19]. A meta-analysis of 103 clinical trials that assessed participant comprehension of the informed consent process and their research involvement found that up to half of participants in these trials did not understand specific components of their informed consent [20]. Informed consent documents are typically lengthy and written above sixth grade reading level, so key information may be difficult to ascertain and the process may prove off-putting for potential participants.

Because enrollment in research studies requires a potential participant to provide consent, an informed consent process that effectively explains the planned research and anticipates questions may mitigate some barriers to research participation and promote better decision-making, potentially leading to more underrepresented patients joining such studies. Recommendations to improve the informed consent process recognize that informed consent is an ongoing process that begins when a potential participant is first approached and continues throughout the study to completion [21–23]. Recommendations are: to tailor consent procedures for specific individuals based on factors such as learning style, language and research literacy; to improve communication skills of research staff; to simplify consent documents for better comprehension; and to utilize electronic consent platforms [21–23]. In 2017, the Federal Policy for the Protection of Human Subjects, also known as the Common Rule, underwent substantive revisions, which included a new informed consent form template. This template highlights key information about the research study at the beginning of the consent form and provides a summary table of research procedures that are part of the study, to provide more transparency [24]. A consent document thus simplified may improve recall and comprehension, whereas additional components of the consent process geared at improving participant comprehension could maximize informed decision-making and study participation.

The Strengthening Translational Research in Diverse Enrollment (STRIDE) study has developed and is testing a multi-component intervention that addresses barriers to research participation during the informed consent process. The integrated, culturally- and literacy-sensitive STRIDE intervention is intended to improve the informed consent process by enhancing its relevance and utility for underrepresented racial/ethnic minority individuals. The primary aim of the STRIDE study is to increase the rate of recruitment (both total number and proportion of enrolled participants) for African American and Latinx participants in ongoing translational research studies at partnering institutions. This manuscript describes the development of the STRIDE intervention.

## Materials and Methods

STRIDE is a multi-site study sponsored by the National Center for Advancing Translational Sciences (NCATS) and the Clinical and Translational Science Awards (CTSA) Program. The collaborating CTSA Program Hubs include the University of Massachusetts Medical School (UMMS), Vanderbilt University Medical Center (VUMC), and the University of Alabama at Birmingham (UAB). The study protocol was approved by the Institutional Review Board (IRB) at UMMS and through reliance agreements with UAB and VUMC.

### STRIDE Intervention Development

STRIDE employed a multi-faceted community-engaged approach to increase relevance and appeal of the intervention components and thus the likelihood of meaningful improvement in the informed consent process. At each study site, we included in our research teams one or more Community Investigators (CIs), local community members of diverse racial/ethnic backgrounds, who contributed to intervention development and pilot testing, and who will participate in dissemination activities. STRIDE includes three components: (1) simulation-based training to enhance research assistants’ cultural sensitivity and ability to engage participants in the eConsent process; (2) an eConsent platform to generate an interactive and engaging informed consent experience; and (3) “storytelling” about the research process to enhance comprehension and cultural relevance of the informed consent and research in general. These components were first developed in parallel using additional community engaged approaches, then assembled together and a pilot test of the integrated intervention was conducted. The development and pilot testing of each of these components is described below.

#### Simulation-based training for research assistants

Onboarding of research personnel is key to efficient and effective conduct of any research study. For example, for clinical trials before recruitment can begin all study investigators and staff are required to complete training modules and demonstrate knowledge of the tools, techniques, procedures, and workflow necessary for the conduct of the study. This training is additive to the general Good Clinical Practice training required by the Office of the IRB, very technical in nature, and unique to each study. Although the informed consent process requires excellent communication skills, communication training for research assistants is not a pre-requisite for initiating a research study. We sought to close this gap by creating an approach to research assistant training is modeled after the simulation-based training approach commonly used in educating medical students and other clinical professions. Briefly, this entails engaging learners in experiences that closely approximate actual practice, providing direct feedback on performance utilizing established standards organized in a checklist and structuring deliberate practice to improve skills.

We based the STRIDE training model on a previously pilot tested protocol and process that was developed at UMass Medical School [25]. This training protocol included engagement of CIs as core design team members partnering on curriculum development, resource creation, and serving as participant-observers during the sessions themselves. CIs advised on all components of simulation curriculum development, case formulation, and checklist refinement with the goal of authentic community representation. They participated in the creation of trigger videos and rating guides to support training of participants and promote interrater reliability amongst CI observers. Prior to the actual simulation training intervention, CI readiness sessions were held to familiarize the community investigators with simulation generally and the STRIDE checklist specifically. Mock consent documents were developed to incorporate e-consent and storytelling components of STRIDE in the simulation training materials.

#### Enhanced eConsent

Details of the development of the eConsent platform, including the technical approach used, have been published elsewhere [26]. The goal of the enhanced eConsent platform was to create an informed consent procedure that could improve a potential research participant’s capacity to comprehend the study and their role as a participant in a way that was literacy appropriate and culturally relevant. The Community engagement studio methodology developed at Vanderbilt University Medical Center was used to develop and refine eConsent components to ensure relevance, usability, and understandability [27,28]. Community engagement studios are facilitated group discussions designed to obtain project-specific input from community members who are the intended recipients of program under development. Studio community experts included African American and Latinx individuals who were recruited through local community organizations in Massachusetts, Tennessee, and Alabama. In each Community engagement studio, a representative of the research team gave a brief presentation about a specific aspect or feature of the eConsent for which feedback was sought (e.g., avatars). A neutral facilitator posed specific questions to the panel of community experts (e.g., lay persons living in the region where the Community engagement studio was conducted) and guided discussion to elicit constructive feedback, whereas a neutral facilitator from VUMC lead the discussion, and a neutral notetaker captured feedback in writing. Feedback was incorporated into the final eConsent elements by the technical team at VUMC.

#### Storytelling videos about research participation

Storytelling was used to provide potential research participants with the opportunity to see and hear from people from diverse backgrounds who had direct experience as participants in past biomedical research projects. This allows presentation of information at appropriate literacy levels using language that is familiar to the target audience [29]. The stories were intended, not to coerce participation, but rather to invite openness and encourage thoughtful review of the research study so that the decision to participate or not is a fully informed one. We used the storytelling production process [30] developed at UMMS to identify storytellers and produce theme-driven, digital-video segments of narratives about the experience of participation in biomedical research. The interview process was directed with a semi-structured interview guide focused on specific domains of interest to potential participants.

We produced transcripts of each interview and performed qualitative thematic analysis using NVivo software [31]. The identified themes were organized into story units, which are stand-alone sections that can join other sections to form complete, coherent narrative segments guided by a prototypical story arc. The interviewer was edited out from raw videos, and each identified narrative stand alone as a story unit. The story units from each storyteller are compiled into an archive, which allows for versatility in the formulation of new content depending on the theme and topic of choice.

Storytellers discussing experiences with research participation were identified in two ways: (1) as a key informant identified by local research teams, and (2) as a storyteller selected from a modified focus group (Story Development Group). The semi-structured interviews with the storytellers were transcribed and thematic analysis was performed to identify themes, which were organized into story units such as “Diversity of biomedical research participation” or “Advice of others considering participation.” These story units were gathered into an archive, which allowed the research team to navigate through different themes connected to different storytellers. To obtain feedback, video samples of each storyteller’s video segments were produced and distributed to the STRIDE CIs (*n* = 4) and to a sample of community members (*n* = 13) identified through a community advisory board to the UMass Center for Clinical and Translational Science. Utilizing an online survey platform, participants for this task were asked to rate the stories for general content, transportation into the narrative [32], or the level of immersion in a story, relevance, and overall satisfaction with the stories presented. The highest-rated stories were included in the STRIDE intervention.

#### Intervention pilot testing

We conducted a pilot test of the consent process utilizing the eConsent and storytelling platforms we developed. The principal investigator of an ongoing clinical research registry at one of the participating institutions agreed to share the associated “live” paper consent document for review by STRIDE CIs and research staff, as well as have an integrated eConsent platform built and shared with participating UAB research assistants/nurses and community members for their input during the piloting phases.

We initially reviewed this exemplar clinical trial’s paper consent document with the STRIDE CIs, a UAB Office of the IRB representative, and the STRIDE research team to assess literacy level, readability, and functionality with the intervention components. Subsequently, changes were instituted to simplify language, reorganize sections, and clarify the potential benefits of the research to future generations. Next, we built the eConsent platform for our exemplar trial by integrating the optimized consent documents within the Research Electronic Data Capture (REDCap) database. The platform was reviewed by seven research assistants/research nurses (five Black, two white) from diverse specialties (e.g., oncology, neurology, cardiology). Feedback about storytelling videos, font size, scrolling features, and videos explaining medical procedures led to revisions to intervention components. Based on the feedback, we developed three versions of the exemplar study eConsent: one for remote review utilizing an avatar in advance of a research appointment (preview mode), one for the participant to review in the presence of a research assistant/nurse during the research appointment (review mode), and one that enabled active consent using the wet signature feature (active full consent mode).

Finally, we conducted mock informed consent experience with potential users of the STRIDE consent platform, with enhancements based on the first phase of feedback to improve the fidelity and feasibility of the intervention. We asked potential users to review the Consent build both remotely and in-person. The purpose of the mock consent procedure was to develop a list of best practices or recommendations for use of the STRIDE integrated intervention platform, which was then used to inform the research assistant simulation training and dissemination phases of the intervention.

## Results

### The STRIDE Intervention

**Fig. 1.**
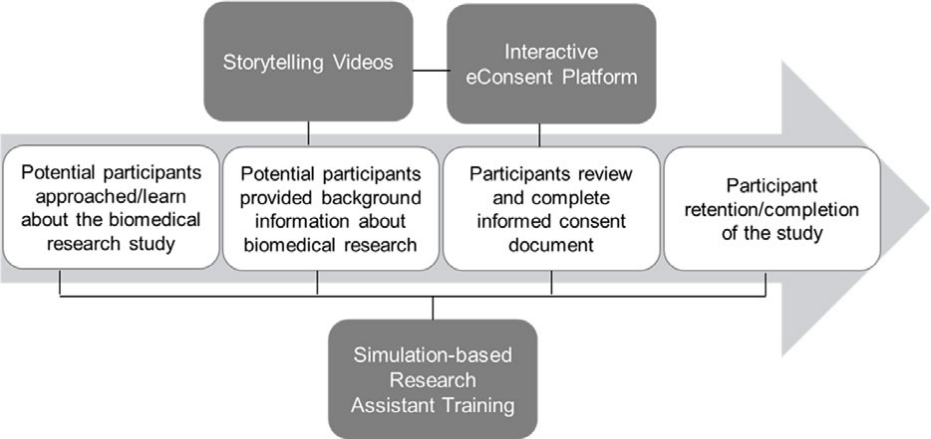
Overview of the informed consent process and the Strengthening Translational Research in Diverse Enrollment (STRIDE) intervention components.

As described above, the final STRIDE integrated intervention consists of three components supporting information exchange during the informed consent process: (1) simulation-based training; (2) an enhanced eConsent platform; and (3) “storytelling” videos about the research process. Fig. 1 describes the aspects of the informed consent continuum that each of the intervention components are intended to address. Each of these three components can be implemented to maximize compatibility with routine study workflow. Although intended to be an integrated intervention, the components can also be used as stand-alone interventions in a research study. The community engaged developmental methods and pilot testing were used to finalize each of the intervention components. A description of each is described below.

#### Simulation-based training for research assistants

The final training protocol includes training scenarios and a series of supporting materials to use in training and evaluation. The protocol includes three scenarios based on actual clinical trial protocols. These scenarios included study interventions with varying degrees of patient risk, time commitment, invasiveness of intervention, and compensation offered. There also was a scenario for situations that would necessitate remote informed consent. Each simulated case involved a potential research participant from a population underrepresented in biomedical research. These cases are to be used for pre- and post-training interactions with the research assistants, as well as for deliberate practice learning experiences for research personnel. The protocol includes a standardized checklist that assesses whether staff communication of informed consent was culturally competent, appropriate to literacy level, and informationally accurate.

Content for research assistant training includes an introduction to simulation-based learning, detailed checklist review, principles of deliberate practice and fishbowl feedback, foundations of cultural humility and competence, and causes and impact of implicit bias on communication and healthcare. Prior to training, research assistants complete a mock informed consent interaction with a standardized patient for a baseline checklist score. After training, research assistants complete or observe two additional standardized patient interactions with deliberate practice, focusing on areas identified for improvement utilizing the standardized checklist to build skills in literacy- and culturally-appropriate informed consent discussion. Research assistants then complete a post-training interaction utilizing the initial mock informed consent case portrayed by a different standardized patient. This model affords both consistency and variety for the scored case. Training occurs in-person over the course of 1.5 days and a refresher training can be deployed as needed (e.g., when new research assistants join research teams).

#### Enhanced eConsent

Details of the STRIDE eConsent component have been published elsewhere [26]. Briefly, we utilized the REDCap platform [33] to create a web-based eConsent platform that includes tools that can be customized for a specific study. This platform facilitates development of study-specific materials that allow potential research participants to select interactive elements and obtain a *personalized* consent experience. These elements include graphical representations of research staff through animated avatars that can guide participants through the consent document, hover-and-click popup definitions of complex terms, and videos depicting procedures that may occur during the research study. Our eConsent portfolio includes a “Wet” signature, which allows capture of voluntary signatures from research participants with a mouse, stylus, or finger. Signatures are captured, stored, and appended as a PNG (portable network graphic) image on the signed PDF (portable document format) document. The eConsent framework can be validated to 21 Code of Federal Regulations (CFR) part-11 compliance and is linked to a central repository able to store and manage version control for consent documents, allowing for informed consent form updates that may occur during a research study.

The goal of the videos included in the video library associated to the e-Consent platform is to familiarize potential participants with the general research process (e.g., randomization) and specific clinical procedures that may occur during a research study (e.g., spinal puncture to obtain cerebrospinal fluid). These videos are 1–4 min long, include voice-over explanation for each procedure described and can be utilized during the consent process at the discretion of a potential participant.

There are several tools (avatars, hover-and-click glossary popups) included in our eConsent platform that support active engagement of potential participants in the informed consent process and that address literacy (and numeracy) and health literacy challenges. For example, when a potential research participant starts interacting with the eConsent platform, they can select graphic representations of research staff as their avatar to guide them through the eConsent document. To provide a more interactive and customized informed consent experience, a participant is able to select the avatar’s appearance and language used by the avatar in their interactions with the participant. When the eConsent document is built the research team can embed scripted messages, which will be delivered by avatar to individuals interested in enrolling in a research study. In addition, to deepen a potential research participant’s understanding of various concepts included in the informed consent, we have incorporated hover/click glossary popups that provide interactive definitions of relevant terms with images, video, or additional text.

Because knowledge about the use of avatar, type of avatar selected, time spent reviewing videos, time spent reviewing various pages of the consent, and use of hover/click glossary popups may be useful to investigators in understanding most commonly utilized eConsent features, the interactions between a research participant and these tools can be tracked using in-line metrics. As part of the informed consent process, the participant is made aware that these metrics are gathered.

The eConsent framework has been designed to function within multiple study/trial workflows and based on the findings from our pilot testing includes a (1) preview mode designed for eConsent dissemination to prospective participants in advance of clinic or study appointments, featuring avatar and in-line popup functionality for consent overview, but without the presence of a study team member or required fields (e.g., wet signature), (2) reviewer mode for in-clinic consent, which allows a member of the study team to quickly highlight/preview the eConsent with a prospective participant without required fields or avatars enabled, and (3) active full consent mode that includes all the features of the eConsent portfolio described above.

#### Storytelling videos about research participation

The storytelling development process yielded 46 unique storytellers, who were individuals who had previously participated in a research study or who had been invited to be in a study but declined participation. Approximately one-third (63%) were female and the majority (70%) were African American, with 11% Latinx and the remainder white.

Through the community engaged story development process, the storytelling videos developed through STRIDE offer potential research participants have the opportunity to see and hear from people with direct experience as participants in past biomedical research projects. Information is presented at appropriate literacy levels and using language that is familiar to the target audience [29].

The thematic analysis resulted in nine unique themes about research participation: (1) historical injustices in research; (2) the importance of research; (3) why diversity in research participation is important; (4) motivations to participate in research; (5) decision-making process about research participation; (6) rights of research participants; (7) experiences with the consent process; (8) experiences interacting with research teams; and (9) advice to people considering research participation. A digital video-archive that includes stories sorted by storyteller and by them has been created. A storytelling toolkit, which includes information on how the stories were created and how they can be used was also developed. The digital stories can be embedded early in the eConsent platform process and may be viewed before a potential research participant begins to review the eConsent content.

## Discussion

In this manuscript, we describe the development and refinement of the STRIDE intervention, an integrated, culturally, and literacy-sensitive, multi-component intervention that aims to address barriers to research participation during the informed consent process. This integrated intervention was developed using multiple community engaged methods to ensure representative community input in addressing barriers to research experienced by underrepresented racial and ethnic minority populations.

This project integrated community engagement into the development and refinement of each of three intervention strategies. Most notably, CIs were important members of the research team. Key steps were taken to ensure the success of this approach. The roles and responsibilities of the CIs were clearly articulated, and they were viewed as experts by the academic members of the team. Each research site employed one or more CI and the CI team formed its own sub-committee within the larger project team. This allowed for networking, opportunities for capacity building, formation of a collaborative identity, and gave collective and individual voices of the CIs more confidence and prominence. In addition, methods to obtain input into selected elements of the STRIDE interventions, such as use of community engagement studios to fine tune elements of enhanced eConsent and community rating surveys to select storytellers, were also used. This allowed for input from a broader range of community members in a highly focused manner. Our approach demonstrated the feasibility and utility of a multi-pronged community engagement approach to ensure that the interventions are relevant and appropriate for their intended audience.

STRIDE attempts to address barriers to research participation through an integrated intervention that includes components that are intended to improve research literacy of potential participants, defined as “the capacity to obtain, process, understand, and act on basic information needed to make informed decisions about research participation.” and improve research team member ability and performance through trainings that challenge assumptions made about racial and ethnic minority member willingness to participation and improved cultural humility and communication skills. By providing a literacy and culturally appropriate informed consent process, STRIDE seeks to address some of the factors that contribute to mistrust in the research enterprise among racial and ethnic minority group members. Although each of the individual elements of the STRIDE intervention addresses aspects of the informed consent process, none addresses the entire process. The STRIDE intervention is an innovative, integrated process incorporating principles of each approach to address the continuum of the informed consent process.

Simulation-based training has become standard practice in medical education and has been shown to improve performance in technical and clinical skills, critical thinking, communication skills and professionalism [34–38]. In STRIDE, our team has applied this approach to training for research assistants, with particular emphasis on providing informed consent to racial and ethnic minority populations. Preliminary results indicate this approach can yield improvements in cultural competency, self-efficacy, and knowledge [25]. The simulation-based research assistant training is intended to challenge assumptions and improve cultural humility and communication skills of research team members, which can, among other things, ultimately improve receptivity to learning more about research. To enhance its scalability and to accelerate dissemination outside our institutions, and to adapt to the necessities of the COVID-19 pandemic, the in-person simulation training we developed as part of STRIDE, has been converted into a virtual program that can be customized to the needs of participating research teams.

Work from our team and others has directly compared eConsent with paper consent and observed that patients and research staff prefer tablet-based eConsent to traditional paper consent [39,40]. A meta-analysis found that multi-media approaches, enhanced consent forms and extended discussions with research staff during the informed consent process can improve participant understanding [41]. Innovative elements of STRIDE eConsent such as hover and click definitions, videos of procedures and read aloud avatars, are intended to provide opportunities to better process and understand what is required in a study.

Communication of health messages through stories is an increasingly popular and effective component of interventions designed to change health behaviors, including in studies conducted among racial and ethnic minority groups [42,43]. Stories may be more emotionally and intellectually engaging than didactic approaches because they present messages within the context of personal experiences and can tap into deep cultural structures, increase cognitive processing and allow for identification with the storyteller [44–47]. The STRIDE storytelling videos present information about research participation in an experiential, rather than didactic format, which is an approach that can improve processing and understanding of information. Learning from storytellers from similar socio-demographic backgrounds can also increase receptivity to learning more about a given research study.

The STRIDE project has limitations that must be considered. It is not possible for a single intervention to adequately address all barriers to research participation. For example, although our intervention approach attempts to address mistrust using a suite of tools that enhance access to research and communication between research staff and potential participant (simulation-based training) and promote informed decision making through improved research literacy (all three interventions), mistrust of research and research participation is a far-reaching phenomenon that will require a comprehensive set of solutions that no single study can fully address. Even though an intervention like STRIDE may improve scientific evidence through better representation in research, it cannot fully address equity when disparities in access to and delivery of health care persist. Future research is needed to test the effectiveness of the STRIDE intervention in increasing representation of African American and Latinx participants in ongoing trials. Our research team is currently conducting a quasi-experimental, interrupted time-series design study to assess this in collaboration with six ongoing research studies.

Although informed consent is considered a longitudinal process that spans the duration of each participant’s continued engagement with the study, the STRIDE intervention targets only the study enrollment/signing of the informed consent document. The intervention was developed with community input from individuals located in the three regions served by the participating academic institutions. However, there may be regional differences in barriers to research participation and preferences for STRIDE intervention components that limit generalizability. Strengths of the STRIDE approach include its community engaged approach; the inclusion of strategies that address participant, research team, and system barriers to research participation; and that the tools developed were designed for broad dissemination throughout the research community.

In conclusion, greater representation in biomedical research is one potential way to lessen health disparities experienced by underrepresented racial and ethnic minority populations by creating direct evidence of efficacy of therapeutics and other interventions in these populations. Much work remains to be done to overcome known barriers and increase participation of these groups in biomedical research studies, and the STRIDE intervention represents a promising building block in this process by integrating a set of tools intended to support efforts to enhance diversity in research projects. The STRIDE intervention will be tested for its real world effectiveness in increasing minority groups’ participation in research and widely disseminated throughout the CTSA consortium and the broader biomedical research community.
